# Preliminary assessment of new single and blended volatile binding media for temporary consolidation of cultural heritage

**DOI:** 10.1038/s41598-024-53828-0

**Published:** 2024-03-01

**Authors:** Hamada Sadek Kotb, Andrea Saccani, Jean-Marc Vallet, Elisa Franzoni

**Affiliations:** 1 Department of Civil, Chemical, Environmental and Materials Engineering, University of Bologna, Via Terracini 28, 40131 Bologna, Italy; 2 Restoration Department, Faculty of Archaeology, Fayoum University, Al-Fayoum, 63514 Egypt; 3 Centre Interdisciplinaire de Conservation Et de Restauration du Patrimoine (CICRP), 21, Rue Guibal, 13003 Marseille, France

**Keywords:** Materials science, Materials chemistry

## Abstract

Volatile Binding Media (VBM) are waxy solids that can be used for temporary consolidation of heritage artifacts and architectural surfaces thanks to their spontaneous sublimation at room temperature. They are used to temporary shelter, consolidate or protect materials during high-risk operation, such as excavation, transportation, water-based treatments, etc. Although they are employed since the 1990s, research focused almost entirely on one of them, cyclododecane (CDD), which is by far the most used in onsite applications. However, CDD exhibits some drawbacks, including a fixed sublimation speed that hardly fits into all the possible applications and climates, hence the development of new VBM is strongly needed. In recent years, a certain attention was addressed to menthol as a possible alternative, but the research on other possible substitutes is still lacking. In this paper, a range of different VBM for temporary consolidation of cultural heritage materials was prepared and investigated, including five pure compounds (CDD, cyclododecanol, cyclododecanone, menthol and camphene) and fifteen mixes. These new materials are expected to provide tunable properties in terms of melting temperatures and sublimation rates, allowing their use in a variety of climatic contexts and applications, and to exhibit better properties for onsite applications compared to CDD, such as lower flash point, lower hazard for conservators’ health and/or higher availability.

## Introduction

The idea of using materials that sublime, i.e., pass directly from solid to gas phase, for temporary consolidation of archaeological artifacts diffused in the 1990s, with the development of the so-called Volatile Binding Media (VBM)^1,2^. These waxy solids can be applied in the melted state or dissolved in suitable solvents and spontaneously disappear with time, with no need of mechanical or chemical treatments, hence they allow to overcome the typical drawbacks of synthetic resins, whose reversibility is known to require extensive use of solvents and to be anyway never complete^1^. VBM can be used for a range of purposes in both archeologic excavations and architectural surfaces: as a consolidant, adhesive and barrier layer^1,3,4^.

The use of VBM as temporary consolidants allows to strengthen artifacts during high-risk operations, exposure to environmental shock and/or transportation to the laboratory, where a proper conservation will be carried out^5^. VBM can be also employed in the block-lifting of archaeological artifacts^6^. Moreover, VBM may be helpful in the detachment of mural paintings, desalination of water-sensitive painted materials, handling of fragile objects, etc.^1–5^. In fact, VBM can be applied to a variety of substrate materials, such as stone, paintings, manuscripts, and textiles^4,7^. The use of VBM was also proposed as adhesive for the installation of temporary sensors^8^.

The first temporary consolidant for conservation was cyclododecane, a saturated hydrocarbon with structural formula C_12_H_24_ (abbreviated CDD), which has been by far the most widely used VBM over the last 20 years^9^. Its properties have been extensively explored^10^, although some limitations were recently highlighted^1^. Firstly, concerns about its toxicity for human health and environment were raised^11^, although a recent paper stated that CDD has no active groups and no health hazards recorded on its Material Safety Data Sheet (MSDS) hence it can be considered not very dangerous^12^. Secondly, it exhibits a low flashpoint, which poses fire risk if temperature higher than 80 °C is reached during melting^13^. Moreover, it is not a strong adhesive or consolidant, resulting useful only in certain treatments^1^. According to some authors, residues of non-sublimated cyclododecane were found in archaeological objects, together with secondary products from its synthesis, but these residues represented < 0.01% of the initial mass of CDD, hence their significance in not fully understood^6^. Finally, CDD provides a single sublimation rate, which is not flexible to address a range of different climates and possible applications. In fact, it was reported to be too slow in certain conditions^1^, and too fast while in others^14^. Different solutions were proposed to speed up or slow down its sublimation, such as the placing of the consolidated objects in warm and ventilated environment or the use of closed containers, respectively^1^, all of which are quite complicated and of course applicable only for small artifacts. Hence, the development of volatile binding media with tunable properties in terms of melting temperature and sublimation speed seems desirable to provide conservators with more healthy solutions and to allow the use of VBM in a variety of different applications and climatic conditions. Unfortunately, a very limited number of publications is available on VBM different from CDD^15^ and little is known about their applications. The most investigated temporary consolidant alternative to CDD is menthol^5,9,11,16^, the main constituent of peppermint oil and widely used for cooling properties, smell, and flavor^17,18^. Menthol has lower melting point and faster sublimation with respect to CDD, being also potentially applicable to moist substrates^9^ but not suitable for very hot climates^10^. Solvent-assisted menthol sols were investigated as temporary consolidant in laboratory and in an archaeological excavation site, showing that the solvent polarity is the most important feature and ethanol, having medium polarity, resulted particularly successful, ensuring good penetration behaviour and good consolidation performances especially in extremely wet condition^19^. The nature of menthol of unsaturated hydrocarbon with an OH group accounts for its good adhesive properties compared to the other VBM but was also suggested to easily oxidize and to give some reactivity with certain kinds of substrates^9^. In the case of the consolidation of a wood ceiling, the acidity of the substrate facilitated a reaction with menthol, resulting in the formation of menthone which failed to sublime completely^20^. However, other authors state that in ambient or cool conditions menthol is most unlikely to react with an ancient surface^11^. Although recent applications are all based on experience^21^, a certain number of studies explored menthol as temporary consolidant, providing an interesting insight on its properties^11,16,22^. In particular, the penetration depth was shown to strongly depend on the viscosity of the melt, which is higher than CDD but also strongly decreasing with temperature^11^. Interestingly, the substrate’s porosity was shown to favour the sublimation speed of menthol, likely due to the higher sublimation surface offered by porous materials compared to compact ones^11^. Other important aspects highlighted were the influence of the solidification speed on the volume shrinkage, which may affect the compatibility of the consolidant with fragile substrates^21,22^. A good compressive strength in combination with sand was found, although depending on both processing procedure and sample size^16^. In general, the solidification of menthol appears quite complicate and seems to involve multiple factors such as solidification transition, thermal diffusion, deformation and stress, all of which may affect the final quality of consolidated samples^21^. Menthol is irritating to mucous membranes^9^, hence it should be preferably used in outdoor and/ or well-ventilated conditions^5^, although some authors consider positively the fact that a well-established safety profile is available due to its long-term usages in different fields^11^. The thermophysical and physicochemical properties of menthol, menthyl lactate and their eutectic mixtures were also studied, finding that the mixes have lower melting points and lower viscosities, hence better penetration and loess lifting ability especially in cold climates, besides allowing to obtain temporary consolidants with melting points in the range of below 0 to 45°C, which conservators can choose from according to their unique application conditions^23^.

To a much less extent, camphene^5,9^ and tricyclene were tested as possible alternative to CDD, although the latter is not available anymore as a product^9^. Camphene occurs naturally in various essential oils from pine trees and can be used as temporary consolidant only in the melted form. It is characterised by low melting temperature and very low flashpoint, quick sublimation and need of stabilization by antioxidants. Its toxicity is reported to be low, although it could cause eye irritation in case of direct contact with eyes^9^.

Setting aside CDD and in part menthol, it is clear that the knowledge about other temporary consolidants is still very limited and much more data should be collected on alternative materials to CDD, especially in terms of sublimation speed, a parameter that is hard to investigate and also to compare in different literature papers^9^.

Because of the shortcoming of CDD, this study aims at exploring alternative VBM for the temporary consolidation of cultural heritage materials, in the preservation of archaeological objects and architectural elements. The selection of the VBM to test was carried out based on their different melting temperatures and sublimation rates, following the preliminary assessment carried out in a previous study^18^. The new VBM investigated in this study are expected:

- to provide a range of melting temperatures and sublimation rates, allowing their use in a variety of climatic contexts and in a range of different applications, thanks to their tunable properties. In fact, the purpose of temporary consolidation may be very different, requiring long- or short-time sublimation speed depending on the situation
- to exhibit better properties for onsite applications with respect to CDD, such as lower flash point, lower hazard for conservators’ health and/or higher availability.

## Materials and methods

### Materials

Five sublimating compounds were investigated in this study:

- Cyclododecane, C_12_H_24_ (labelled as CDD). According to the datasheet of the product, its density is 0.82 g/cm^3^ and its melting temperature is 63 °C. Flash point: 98 °C^1^
- Cyclododecanol, C_12_H_24_O (labelled as CDNOL). According to the datasheet of the product, its density is 0.9 g/cm^3^, its melting temperature is 76–79 °C and its flash point is 138°C
- Cyclododecanone, C_12_H_22_O (labelled as CDNONE). According to the datasheet of the product, its density is 0.9 g/cm^3^, its melting temperature is 62 °C and its flash point is 118 °C
- Camphene, C_10_H_16_. According to the datasheet of the product, its density is 0.84 g/cm^3^, its melting temperature is 48–52 °C and its flash point is 26 °C
- Menthol, C_10_H_20_O. According to the datasheet of the product, its density is 0.91 g/cm^3^, its melting temperature is 32–36 °C and its flash point is 92 °C.

These five compounds were subjected to some basic characterisation in a previous study^24^. The results highlighted that they exhibit a very different behaviour, hence they were selected for further testing, alone and in blends, in this paper, with the aim of developing a range of VBM with tunable properties. In brief^24^, they not only exhibit a wide range of melting temperatures (from 30 to 77 °C) and relevant crystallization temperatures (from 17 to 73 °C), but also very different sublimation speeds, which strongly depend on temperature too. Camphene has an extremely high sublimation speed even at 20 °C (about 20 times higher than CDD), probably related to its amorphous or fine crystalline structure, hence its use seems possible only for very cold climates and/or very fast operations. Menthol exhibits a very low melting temperature and a slow solidification (long workability), suitable for cold climates, besides a slightly faster sublimation than CDD. CDNOL is much slower in sublimating than CDD, hence it seems suitable for very hot climates. CDNONE, despite exhibiting a thermal behaviour basically overlapping that of CDD, sublimates considerably slower than it.

In terms of flash point, CDNOL and CDNONE exhibit a sensibly higher temperature than CDD, hence they involve a decrease of fire risk in archaeological excavations. The flash point of menthol is only slightly lower than CDD, hence they appear basically comparable. Camphene has a much lower flash point than CDD and might arise flammability risk also at environmental temperature. However, camphene was selected for testing for its very quickly sublimation rate, hence it was considered interesting especially as a component to prepare mixtures with other VBM, aiming at speeding up their sublimation rate. When used in blends, camphene is expected to exhibit a more affordable flash point, which in any case will have to be determined.

In this paper, not only pure VBM were tested, but 15 mixes were also prepared and characterised, whose labels and compositions are reported in Table 1. In general, the mass ratios explored were 25:75, 50:50, 75:25, but not all the possible combinations were tested, because attention was given to the mixes regarded as most significant and promising. The melting of the mixes was carried out in an electrically heated wax melter used in the batik and encaustic art crafts. The tool consists of a “pen” equipped with a brass pan for melting and a drain channel for pouring the liquid wax. In the melting of the mixes, particular care was addressed to stir the liquid, to obtain a uniform mix. Some of the mixes were discarded, due to their defective workability in the melt state and/or unproper aspect when solidified (e.g., sticky, non-uniform, etc.), hence they were not subjected to further testing. The mixes selected are the ones in grey in Table 1.

**Table 1:** Mixes of VBM under investigation in this study (mass %).

Mix name	Camphene	CDD	CDNOL	CDNONE	Menthol
**M1**	**–**	**50**	**50**	**–**	**–**
M2	50	–	50	–	–
**M3**	**25**	**–**	**75**	**–**	**–**
M4	–	–	50	–	50
**M5**	**–**	**–**	**75**	**–**	**25**
M6	–	50	–	–	50
**M7**	**–**	**75**	**–**	**–**	**25**
**M8**	**–**	**75**	**25**	**–**	**–**
M9	50	–	–	50	–
M10	25	–	–	75	–
**M11**	**–**	**–**	**25**	**75**	**–**
**M12**	**–**	**–**	**50**	**50**	**–**
**M13**	**–**	**50**	**–**	**50**	**–**
M14	–	–	–	50	50
M15	–	–	–	75	25

The ones with bolded are those selected for the following tests.

The behaviour and properties of the pure VBM and their mixes were firstly investigated. Solid pieces of the different consolidants were used, after melting and cooling, for some characterizations, while in other cases, the melted consolidants were applied over the following substrates:

- Laboratory glass slides
- Carrara marble slabs (supplied by Imbellone Michelangelo s.a.s., Bologna, Italy), quarried in the Apuan Alps in Italy. This stone is mainly composed of calcite (98%), with small traces of dolomite, and exhibits an extremely low open porosity (2.3%)
- Porous limestone, quarried in the Lecce area in Italy (Cursi-Zollino-Melpignano quarry) and provided by Décor s.r.l., San Giovanni in Fiore, Italy. This is a highly porous organogenic calcareous stone, Lecce stone, mainly composed of calcite (86%), with traces of quartz and phosphatic minerals, and having an open porosity equal to 37.7%.

The application on substrates was carried out to investigate the possible role of the material in the sublimation of the consolidants. Testing the VBM over laboratory glass slides and Carrara marble slabs, both exhibiting basically no porosity and composed of silica and calcite, respectively, allowed to specifically investigate the influence of the chemical composition of the substrate on the sublimation rate. In fact, although glass considerably differs from real inorganic substrates, which usually contain crystalline silica and are characterised by surface roughness and deterioration patterns, it allowed to test whether some bonding forms with SiO_2_ which may not form with CaCO_3_. Conversely, the role of the substrate porosity was investigated comparing the VBM behaviour over marble and limestone samples, both exhibiting a major CaCO_3_ composition but having negligible porosity and high porosity, respectively.

### Methods

The melting and crystallization temperatures were investigated by Differential Scanning Calorimetry (DSC). These temperatures are key parameters to assess the applicability of the VBM in different climates, their compatibility with fragile organic substrates, and their thermal behaviour. DSC was performed in a TA Instruments DSC Q10 model with a Refrigerated cooling system 90. The sample (mass 5–10 mg) was put in the aluminium pan and covered, and the analysis was performed in N_2_ flow (20 mL/min) at heating/cooling rate 5 °C/min. Two heating and cooling cycles were performed. While the first cycle (cycle 0) was aimed at eliminating the thermal history of the samples and hence was discarded, the following cycle was used for this study, as it was considered representative of the thermal behaviour of the compounds, independently on their thermal history due to manufacturing and any spurious effect due to preparation and stocking.

The possible formation of new phases in the mixes was investigated by Fourier Transform Infrared Spectroscopy (FT-IR), in a Perkin Elmer Spectrum One spectrometer equipped with a diamond crystal in attenuated total reflectance (ATR) mode, which was used to collect data in the region 4000–400 cm^−1^ (8 cm^−1^ resolution, 16 scans).

The crystallographic structure of the VBM was investigated by X-Ray Diffraction (XRD), in a Pananalytical EMPYREAN instrument (Cu-Kα, 40 mA, 2θ = 5°–60°). The samples were melted and placed directly on the holder, performing the analysis immediately after cooling. The crystalline vs. amorphous state may influence the sublimation speed of the compound and possibly also its volume change during solidification, although this parameter was not specifically investigated in this study.

The sublimation speed of the products was investigated by two different techniques. Thermogravimetric analysis (TGA) was used to investigate the sublimation speed of the VBM in controlled conditions, at different temperatures. The collection of data in controlled condition was considered important for a first evaluation and comparison of the different VBM, given the difficulties in assessing this parameter in a comparable way^9^. The analysis was carried out in a Perkin Elmer TGA 7 instrument on samples of 12 ± 2 mg, starting from laboratory temperature (~ 20 °C), in a nitrogen flow (40 mL/min). Samples were subsequently heated at 30 °C, 40 °C, 50°C, 60 °C, 70 °C, and 80 °C, keeping the temperature constant at each step for 15 min, to determine the weight loss over time. Fifteen minutes were assessed to be enough to have a clearly linear mass loss. The maximum temperature for each material was selected based on its melting point.

Moreover, the melted VBM were poured over the three selected substrates (size of the laboratory glass slide: 26 mm × 75 mm, size of the marble and limestone samples: 2.5 × 2.5 × 1 cm^3^), keeping the area of coverage almost the same (diameter of ~ 2 cm), as well as the amount of VBM deposited (0.25 ± 0.05 g). Then, the samples were put in different environments:

- Laboratory environment at 20 ± 5 °C, by leaving the samples in a fume hood
- Laboratory environment at 30 ± 5 °C, by keeping the samples in a ventilated climatic chamber.

The sublimation speed of the different VBM was monitored by periodically measuring the mass loss of the samples.

## Results and discussion

**Figure 1:**
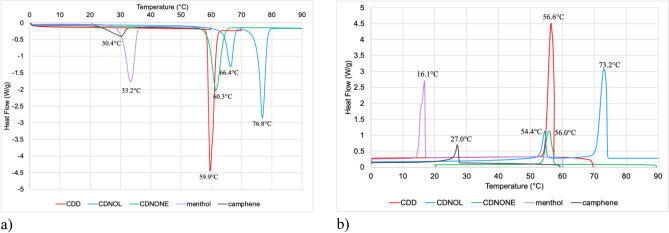
DSC curves of individual VBM during heating (**a**) and cooling (**b**).

The DSC curves of the single VBM are reported in Fig. 1, while the melting and crystallization temperatures found by DSC are collected in Table 2.

**Table 2:** Melting and crystallization temperatures found by DSC for the different VBM.

	CDD	CDNOL	CDNONE	Menthol	Camphene	M1	M3	M5	M7	M8	M11	M12	M13
T_melting_ (°C)	60	62; 77	60	26; 33	30	62	67; 80	60	13; 55	63	59	59	59
T_cryst._ (°C)	57	73; 54	56	16	27	58	58; 76	53	50	57	55	55	52

In the case of CDD and CDNONE, the melting temperature (Fig. 1a) was in line with what reported in the technical datasheets. Considering that the fusion enthalpies and the densities of CDD and CDNONE are very similar (fusion enthalpy 14.8 kJ/mol for CDD^25^ and 16.8 kJ/mol for CDNONE^26^; density 0.82 kg/L for CDD and 0.90 kg/L for CDNONE), the fact that the peak of CDNONE is much smaller than that of CDD, could be due to the presence of an amorphous fraction in CDNONE. The main peak of menthol is at the temperature reported in the datasheet, as well. For CDNOL, the main melting point is in line with what reported in the datasheet, but a secondary peak is present at 66.4 °C, which could be due to some residual CDD left in the product after the CDNOL synthesis and finely intermixed with the main CDNOL structure. In the case of camphene, a melting peak was observed at 33.2 °C (starting from about 20 °C), i.e., almost 20 °C lower than the value reported in the datasheet, and the area of this peak is very small, suggesting a very limited crystallinity of the material.

**Figure 2:**
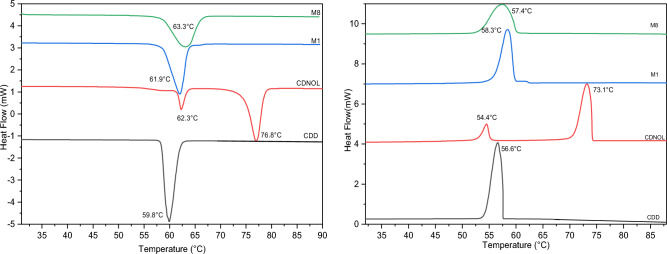
DSC curves of M1 and M8, together with their pure components CDD and CDNOL (left: heating, right: cooling).

During cooling (Fig. 2b), crystallization occurs at a temperature about 3 °C lower than melting for CDD, CDNONE, camphene and CDNOL (major peak). Conversely, a strong undercooling (about 17 °C) is necessary for the crystallization of menthol, suggesting that this material has a longer workability but is also suitable for cold climates only. Also for the minor peak of CDNOL, tentatively ascribed to residual CDD, a strong undercooling is necessary (12 °C), probably because this secondary phase is trapped inside the CDNOL microstructure.

**Figure 3:**
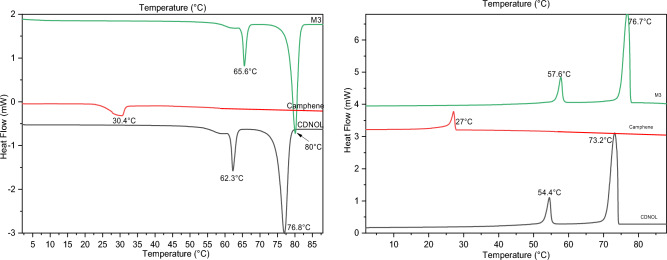
DSC curves of M3, together with its pure components camphene and CDNOL (left: heating, right: cooling).

**Figure 4:**
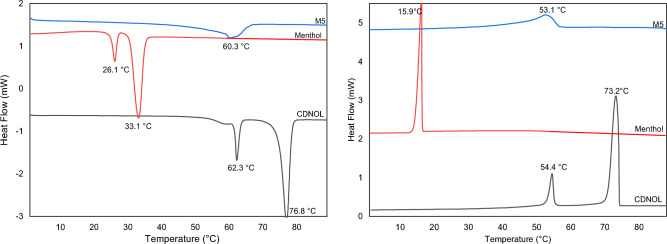
DSC curves of M5, together with its pure components menthol and CDNOL (left: heating, right: cooling).

**Figure 5:**
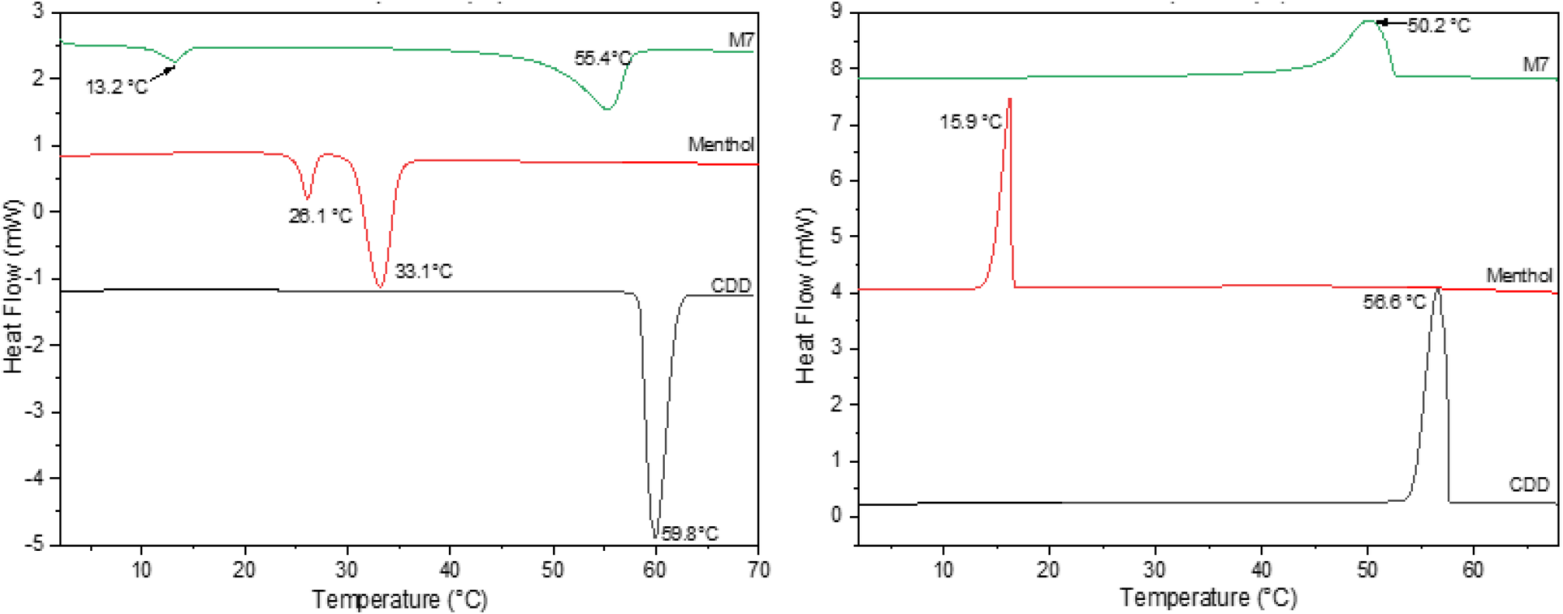
DSC curves of M7, together with its pure components menthol and CDD (left: heating, right: cooling).

**Figure 6:**
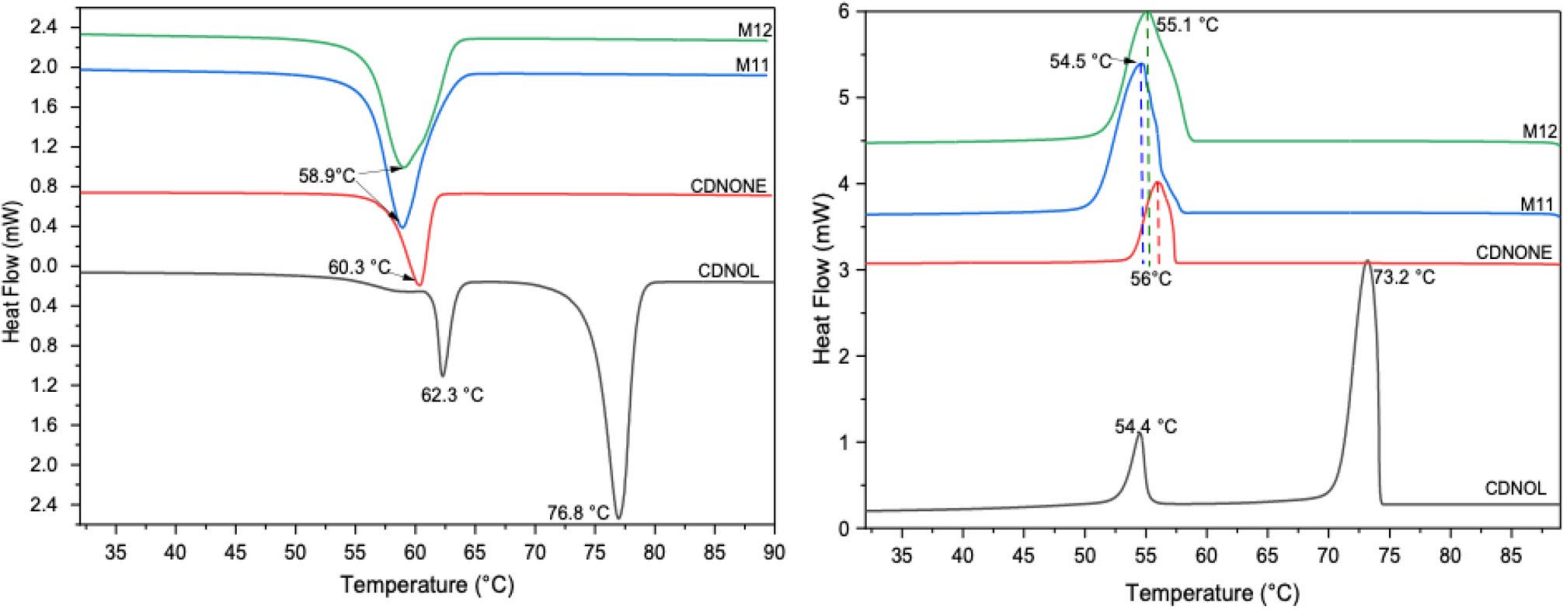
DSC curves of M11 and M12, together with their pure components CDNOL and CDNONE (left: heating, right: cooling).

**Figure 7:**
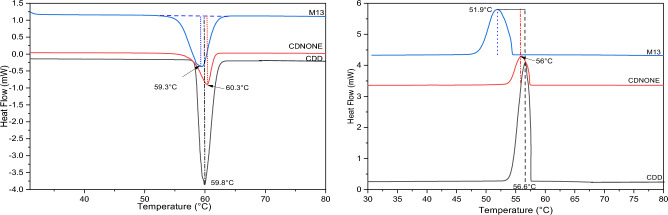
DSC curves of M13, together with its pure components CDS and CDNONE (left: heating, right: cooling).

Figures 2, 3, 4, 5, 6 and 7 report the DSC curves of the VBM mixes, in comparison with those of the pure compounds, the latter ones analysed as supplied by the manufacturers, i.e., in the condition in which they would be used by conservators. The melting and crystallization temperatures found by DSC are reported altogether in Table 2. The results of DSC analysis of the mixes allow to make the following observations:

- M1 and M8, obtained by mixing CDD and CDNOL in different proportions, exhibit one single peak both in heating and in cooling, approximately corresponding to the peak of CDD, while the main peak of CDNOL seemingly disappears (Fig. 2). As the CDNOL fraction cannot have sublimed during the time of the test, being the slowest among all the VBM (see below), this can be ascribed to an impossibility for CDNOL to crystallize in presence of CDD
- M3, made mixing CDNOL and camphene, exhibits a thermal behaviour basically overlapping CDNOL, with only a slight increase (about + 3 °C) in the temperatures of melting and crystallization (Fig. 3). Considering that the peak of pure camphene is very small and that the amount of camphene in M3 is just 25%, this result seems consistent with the material’s formulation
- M5, obtained from menthol and CDNOL, exhibits a peculiar curve, with only a small peak which does not correspond to the main peak of CDNOL neither to that of menthol (Fig. 4). This peak is very close to that of CDD, being possibly due to the CDD impurities in CDNOL, hence it seems that an amorphous mass formed with only small crystals ascribed to the impurities
- Also in the case of M7, obtained from menthol and CDD, the mix exhibits both in heating and cooling only one peak, which is at an intermediate temperature between the melting/crystallization temperatures of the two pure constituents (Fig. 5), possibly suggesting the formation of an amorphous mass with some limited defective crystals having peaks at 13.2°C and 55°C. The very small peak at 13.2°C disappears during cooling, likely due to sublimation
- M11 and M12, obtained mixing CDNOL and CDNONE in different proportions, exhibit a behaviour like what observed for M1 and M8, where CDNOL was mixed with CDD (Fig. 6). In fact, also in this case the main peak of CDNOL disappears in the mixes, and only the peak pf CDNONE (very similar to CDD, by the way) remains
- in the case of M13, given the similarity between the thermal behaviour of the two components of the mix (CDD and CDNONE), the DSC curves show basically the same trend, with only a lower crystallization temperature with respect to the pure components, possibly beneficial for onsite applications (Fig. 7).

**Figure 8:**
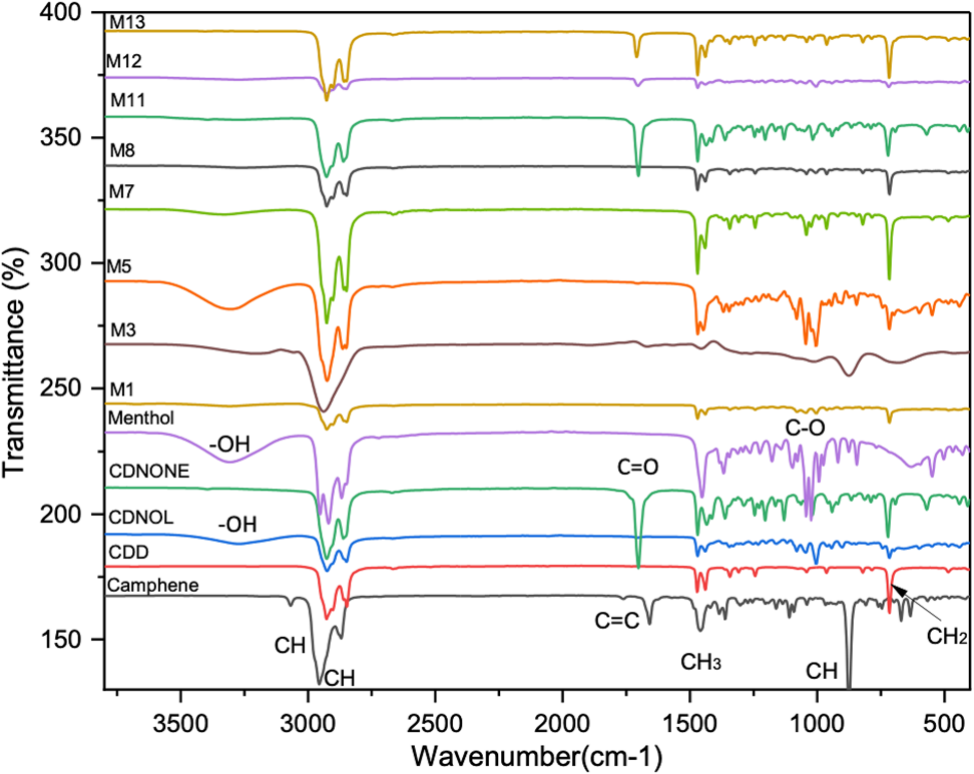
FT-IR spectra of the pure VBM and selected mixes.

The FT-IR curves of the pure VBM and their mixes are reported in Fig. 8. The results indicate that no new compound formed and that the mixes simply contain the bands present in the relevant pure components.

**Figure 9:**
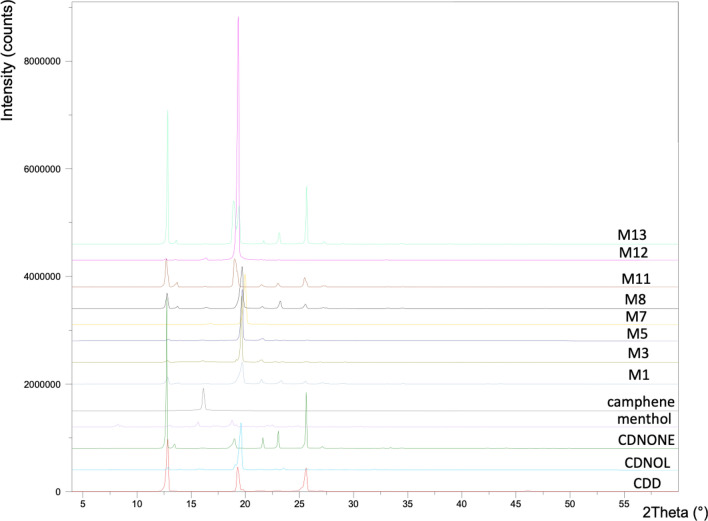
XRD spectra of the pure VBM and selected mixes.

The XRD spectra of the pure VBM and selected mixes are reported in Fig. 9. It is noteworthy that the consolidants investigated by XRD cooled down in laboratory conditions, hence they solidified not necessarily at the same speed used in DSC analysis (cooling rate 5 °C/min), which may have led to a different size of the crystallites. Concerning the pure VBM, the curve of menthol exhibits low and quite broad peaks, suggesting a small size of crystallites according to Scherrer’s theory. The dependence of the menthol’s crystallite size was highlighted also elsewher^16^. In the case of camphene, the single and quite small peak suggests a low crystallinity and/or small crystallites’ size. The diffraction patterns of CDD and CDNONE exhibit some similarities, having three peaks aligned, although with different heights, however the diffraction pattern of cyclododecanone seems still to be explored in consideration of its possible different conformations^27^. The spectrum of CDNOL exhibits basically one strong peak only, suggesting a small size of the crystallites, with only marginal peaks ascribable to the CDD impurities already detected above. Concerning the mixes, the results are not easy to interpret, due to the proximity of some peaks of the different pure VBM. In the case of M1 and M8, it is difficult to confirm the disappearance of CDNOL in the crystalline form suggested by DSC, as pure CDNOL exhibits basically only a single strong peak that almost overlaps one major peak of CDD. The diffraction pattern of M3 is consistent with what found by DSC, as only the peaks of CDNOL can be found while the peak of camphene is almost negligible. The curves of M5 and M7, which are mixes with menthol, are hard to interpret, due to the low diffraction pattern of menthol and the presence of a single peak in the region around 2θ = 18.5°. In the case of M11 (CDNONE:CDNOL 75:25) only the peaks of CDNONE are visible, while in the case of M12 (CDNONE:CDNOL 50:50) a single major peak seems present making conclusive observations impossible. M13 exhibits the peaks of both pure VBM from which it was obtained, as expected.

**Figure 10:**
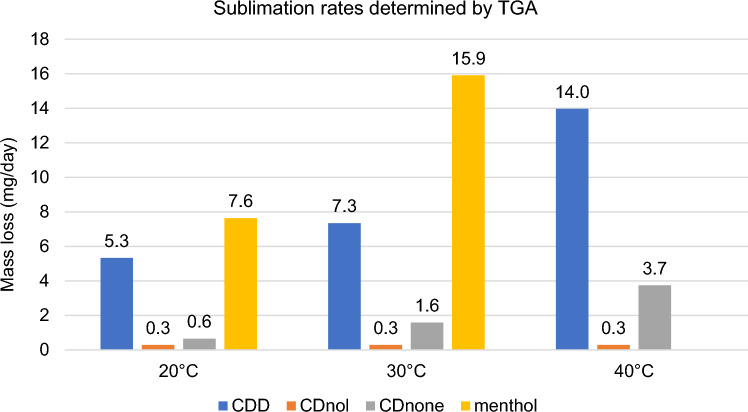
Sublimation rates of the pure VBM found by TGA.

**Figure 11:**
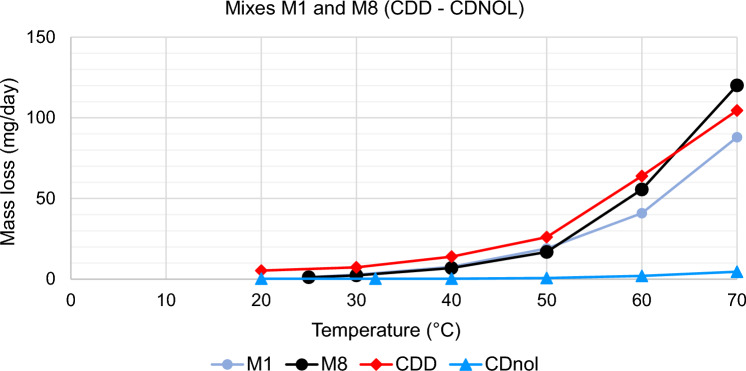
Sublimation rates of M1 and M8, together with their pure components CDD and CDNOL.

**Figure 12:**
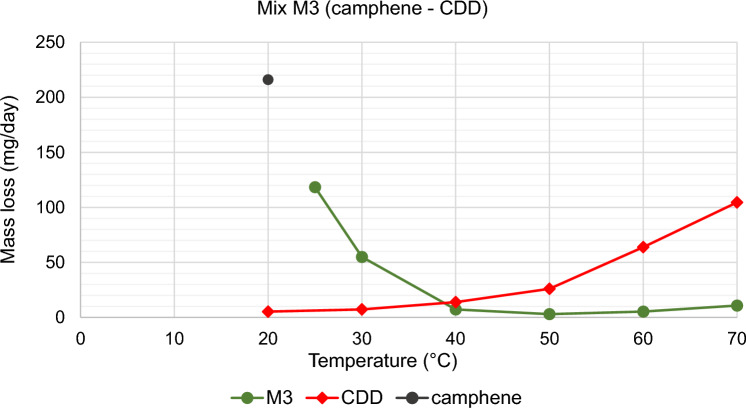
Sublimation rate of M3, together with its pure components CDD and camphene.

**Figure 13:**
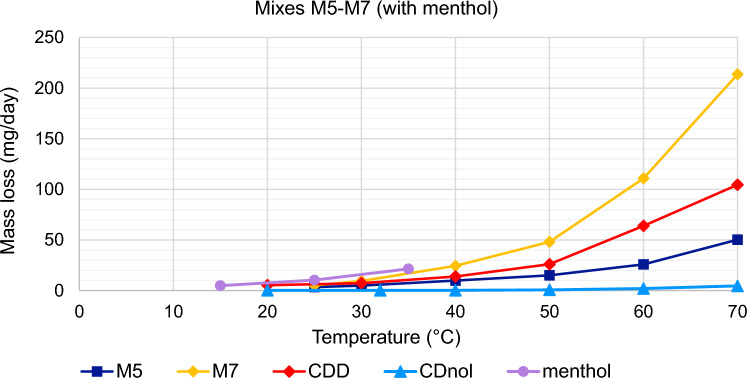
Sublimation rates of M5 and M7, together with their pure components CDD, CDNOL and menthol.

**Figure 14:**
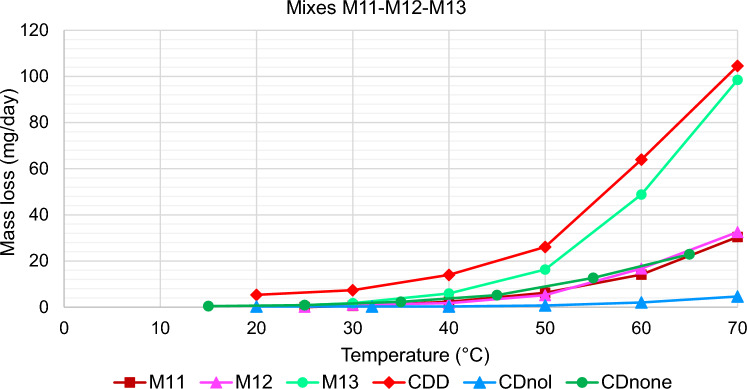
Sublimation rates of M11, M12 and M13, together with their pure components CDD, CDNOL and CDNONE.

**Figure 15:**
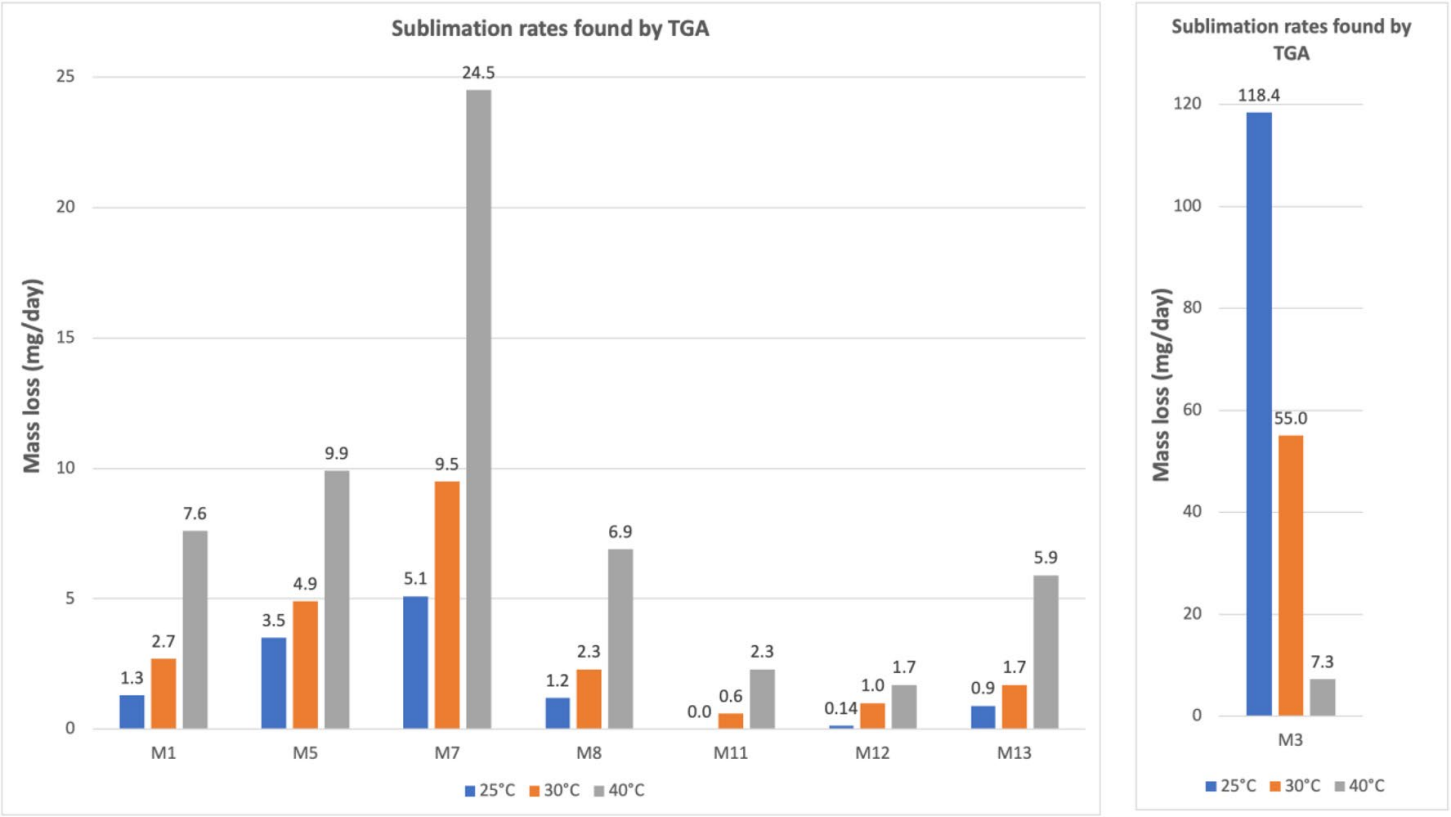
Sublimation rates of all the mixes found by TGA at 20 °C, 30 °C and 40 °C.

**Figure 16:**
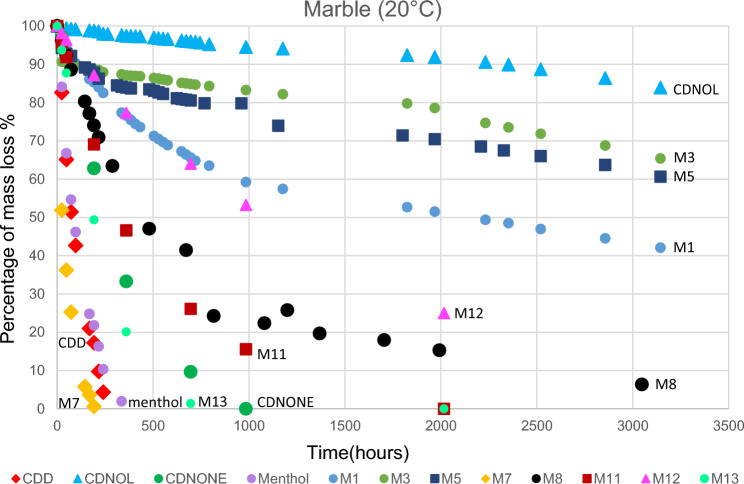
Sublimation rates of all the mixes applied over marble substrate, at 20 °C.

**Figure 17:**
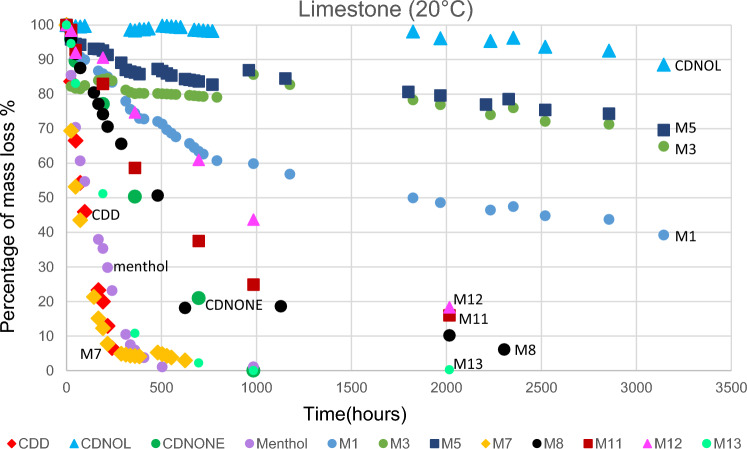
Sublimation rates of all the mixes applied over limestone substrate, at 20 °C.

**Figure 18:**
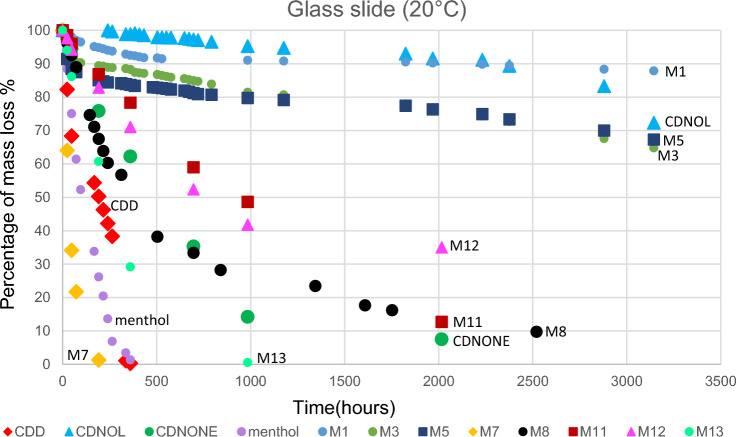
Sublimation rates of all the mixes applied over glass slide substrate, at 20 °C.

**Figure 19:**
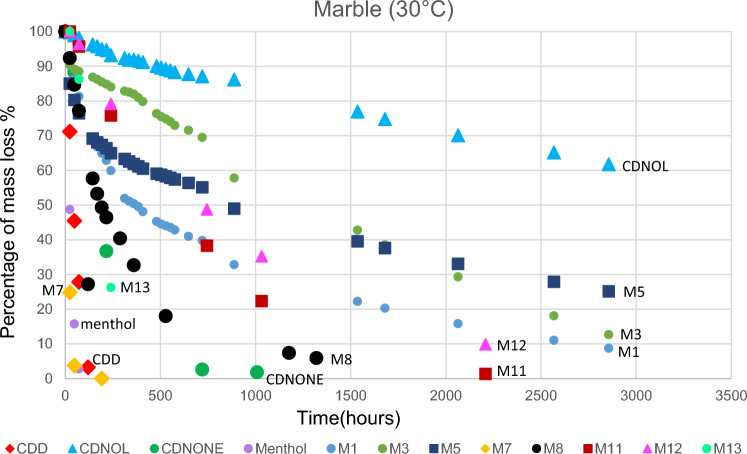
Sublimation rates of all the mixes applied over marble substrate, at 30 °C.

**Figure 20:**
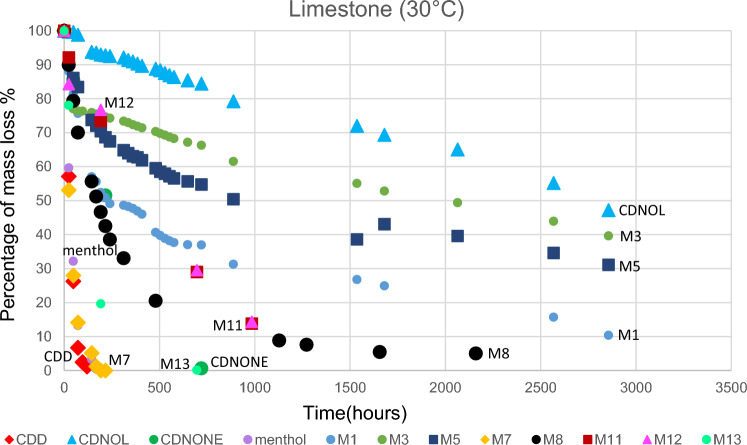
Sublimation rates of all the mixes applied over limestone substrate, at 30 °C.

**Figure 21:**
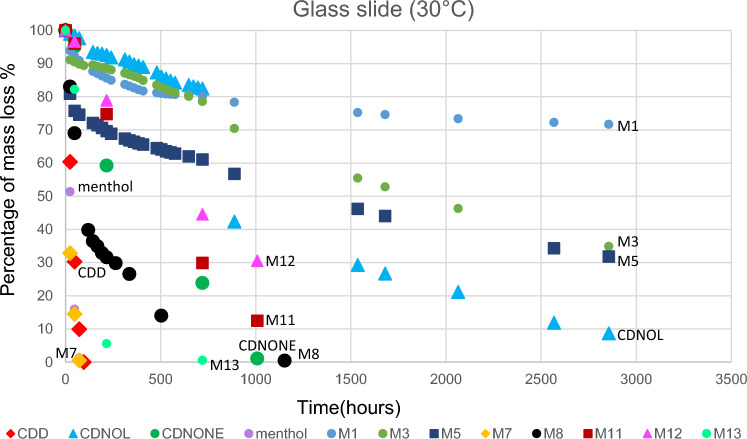
Sublimation rates of all the mixes applied over glass slide substrate, at 30 °C.

The results of TGA are reported in Figs. 10, 11, 12, 13, 14 and 15. In particular, the sublimation rates of the pure VBM and mixes at different temperatures are reported in Figs. 11, 12, 13 and 14, while the relevant values at 20 °C, 30 °C and 40 °C, i.e., the most significant temperature for in-the-field applications, are summarised in Figs. 10 and 15. Afterwards, the sublimation rates of pure VBM and mixes are reported in the case of their application over standard substrates (marble, limestone and glass) and exposure to air at 20 °C (fume hood) and at 30 °C (climatic cabinet) (Figs. 16, 17, 18, 19, 20, 21). Observing and comparing the results of the sublimation rates of the pure VBM in different conditions, the following remarks can be done.

- Analysed by TGA, camphene exhibited the highest sublimation speed, namely 216 mg/day at 20 °C, which is 1–2 orders of magnitude higher than the speeds of the other VBM. It was not possible to determine in a reliable way the sublimation speed at higher temperatures, due to the quick loss of the sample during the analysis. When applied to standard substrates, it completely disappeared in about one day, hence the relevant data are not reported in the figures.
- Menthol is the fastest among the other pure VBM, followed by CDD, which is only slightly slower (Fig. 10). Menthol is highly sensitive to temperature, likely due to its low melting point (33 °C), and in fact its sublimation rate approximately doubles passing from 20 to 30 °C. CDD is sensitive to temperature too, although to a minor extent than menthol. When applied over the substrates, both menthol and CDD are completely lost in about 12 days at 20 °C, while this time drops by half at 30 °C (Figs. 16, 17, 18, 19, 20, 21), suggesting that in hot climates these two consolidants are suitable only for short-term operations (few days). The nature of the substrate seems not to play a key role in their sublimation rates. Considering that the amount of VBM deposited over the standard substrates was about 0.25 ± 0.05 g, the daily loss of CDD and menthol at 20 °C can be estimated in about 20 mg/day, a value which is higher than that found by TGA (5.3 and 7.6 mg/day, respectively, Fig. 10), but in the same order of magnitude. The difference seems related, on the one hand, to the higher air exchange occurring on the samples located in the fume hood and climatic chamber with respect to those inside TGA and, on the other hand, to the fact that nitrogen was used as the purge gas in TGA rather than air. However, the results indicate that TGA is reliable for collecting general data on the sublimation speed of VBM, even if the real speed is obviously influenced by multiple factors.
- CDNOL is the slowest among all the VBM, probably as it exhibits the highest melting temperature, notwithstanding its apparently scarce crystallinity or small crystallites’ size. Moreover, according to TGA (Fig. 10), its sublimation does not change in the range 20–40 °C, suggesting a low influence of temperature. In the tests with standards substrates, CDNOL confirms to be the slowest VBM, but a higher effect of temperature was observed. Over marble, after 100 days, it reached about 88% of its initial mass at 20 °C and 64% at 30 °C. Over limestone, the effect of temperature was even stronger. In the case of glass at 30 °C, the sudden mass loss at about 100 h experienced by CDNOL (Fig. 21) was probably due to the detachment of a fragment, hence it was not considered. Hence, the comparison among the different substrates did not highlight systematic differences in the sublimation speed of CDNOL. Actually, a certain interaction between the -OH groups of CDNOL and glass could be expected, but probably the consolidant layer is so thick and the sublimation speed is so low that this interaction becomes significant only when the consolidant is almost completely disappeared.
- Despite the similarity of CDNONE with CDD in terms of melting/crystallization temperatures (Fig. 7) and diffraction patterns (Fig. 9), CDNONE resulted slower in sublimating than CDD, although faster than CDNOL. While in TGA its sublimation rate seems more similar to CDNOL than to CDD, the results obtained over standard substrates clearly indicate that the loss of CDNONE is not so slow, being completely lost over carbonate substrates in about 1000 h at 20 °C and 750 h at 30 °C. Interestingly, the speed of sublimation over glass is considerably lower at both temperatures, indicating a certain role of the substrate composition, possibly owing to the interaction between the C=O group of CDNONE and the -OH on the glass.

In the case of the mixes, the following remarks can be done.

- M1 and M8, obtained by mixing CDD and CDNOL in proportions 50:50 and 75:25 respectively, exhibited an intermediate behaviour when analysed by TGA, and the sublimation speed is higher for the mix with the highest CDD content, as expected. This seems to suggest that the combination of these two VBM with very different sublimation speeds may allow the formulation of temporary consolidants with tunable sublimation. The same intermediate behaviour was observed when the products were applied over standard substrates and left at 20° and 30 °C. However, the curves of mass loss clearly indicate the presence of two distinct slopes (higher in the first part of the curves and lower in the second part), ascribed to the separated sublimation of the CDD and CDNOL fractions, respectively. This aspect was not detected by TGA, which allows to measure only the initial mass loss speed. Concentrating on the carbonate substrates, in the first ~ 10–20 days, the CDD fraction is lost, at a speed with is slower than pure CDD likely due to the presence of a fine mixture, and afterwards the CDNOL fraction is lost at a much smaller speed. This is very important, because it suggests that a suitable time must pass before the residues of the consolidant are completely lost and further treatments can be applied. When M1 is applied over glass slide, its sublimation becomes much slower and similar to CDNOL alone, which is difficult to explain and is still under investigation, because this effect was not observed in the two starting VBM.
- M3, in which the fastest and the slowest VBM where mixed (CDNOL:camphene 75:25), exhibited an interesting behavior, as it is very clear that camphene quickly sublimes, before CDNOL. In TGA, where the material was exposed subsequently to increasing temperature, the mass loss was quick at the beginning and slower afterwards, simply because the camphene had been already progressively lost. This leads to an apparent decrease of the sublimation speed passing from 20 to 40 °C in Fig. 15. Over the substrates, the loss of the camphene fraction is even more recognizable, as a fast loss of about 25% of the initial mass occurs at the beginning of the test.
- M5 is again a mix of a fast and slow subliming compounds (CDNOL:menthol 75:25) and also in this case the sublimation speed is intermediate, and the sensitivity to temperature of menthol is mitigated by the presence of CDNOL (Fig. 13). Over the substrates, also in this case the slope of the curves is not linear, being faster at the beginning (especially at 30 °C). However, especially at 20 °C, there is not a drastic separation between menthol and CDNOL sublimation, unlike the previous mix, suggesting a higher degree of interaction between the two components.
- In the case of M7, where CDD and menthol were mixed, TGA highlighted that the sublimation speed was very high, and strongly increasing with temperature (Fig. 13). Over the substrates, M7 is the fastest among all the mixes, being lost in a time from few days up to one week for marble and glass (of course, more quickly in the case of 30 °C) and a bit more slowly on limestone. This is the first case in which the porosity of the substrate seems to affect the sublimation speed, as expected, suggesting some degree of capillary absorption of the consolidant.
- M11 and M12, obtained by mixing CDNONE and CDNOL in proportions 75:25 and 50:50 75:25 respectively, exhibited a very interesting behaviour (Fig. 14). Their thermal behaviour investigated by TGA was intermediate between the pure VBM, as in other cases before. However, the mass loss curves do not show any change in the slope, suggesting that the sublimation is concurrent. The sublimation test over the substrates gave higher values than those found by TGA, consistently with what already highlighted for CDNONE. The sublimation was faster on marble than on limestone, as expected based on the porosity of the latter one, suggesting some possible penetration, while the comparison between glass and marble gave not conclusive results on some significant interactions.
- M13, obtained mixing equal masses of CDD and CDNONE, exhibited intermediate behaviour among the two pure VBM, both in the TGA and in the test of sublimation on the substrates. These results are consistent with the behaviour of the single VBM and with what expected, also in terms of shape of the curve.

## Conclusions

In the present paper, temporary consolidants alternative to CDD, including pure compounds and mixes, were prepared by melting, characterised and compared with CDD. Their thermal behaviour, interactions, crystalline structure and sublimation speed with and without substrates were investigated. The results allowed to derive the following conclusions.

- The melting temperatures of the investigated consolidants are comprised between 30 and 77 °C, which represents a wide enough range to match different climatic conditions. Although for organic materials, such as wood, paper and textile, VBM are usually required not to exceed the melting temperature of 65 °C, the maximum temperature found in this study, namely 77 °C, can still be considered acceptable for some inorganic substrates.
- All the crystallization temperatures are about 3 °C lower than the melting ones, except for menthol, whose crystallization occurs at about 17 °C less than melting, suggesting a slow solidification and a suitability mainly for cold climates.
- Menthol and CDNOL exhibited double peaks in DSC analysis, likely owing to impurities in the product (recognizable as CDD in the case of CDNOL). The impurity of menthol exhibited a melting temperature lower than the value declared in the technical datasheet.
- According to FT-IR, no new phases formed from chemical reactions in the investigated mixes, which were prepared and applied in the melted state. In some cases, an intimate mix or an amorphous mass formed by combining some of the VBM, while in others the two components remained clearly separated and distinguishable during the characterization tests. When an intimate mix was obtained, the consolidant exhibited intermediate properties among the starting VBM.
- The XRD analysis suggested a different degree of crystallinity for the different consolidants, but it resulted hard to interpret, mainly due to its sensitivity to the crystallites size, which in turn depends on the solidification speed, which was not taken into specific account in this study.
- In the tests by TGA, camphene exhibited an extremely high sublimation speed, i.e., one order of magnitude higher than the others. The other consolidants exhibited different sublimation speeds, according to the ranking: menthol > CDD > CDNONE > CDNOL. The sublimation speed resulted strongly dependent on the temperature, especially for materials with low melting point, while for CDNOL, having the highest melting point, it was less affected by temperature.
- In presence of substrates, the sublimation speed was generally higher than in TGA, likely due to the bigger air exchange in open conditions. The porosity of the substrate seems to play a role in slowing down the sublimation, as expected, but only for some consolidants, which were probably able to penetrate for a certain depth into the substrate. The nature of the substrate (calcite, silica) influenced in some cases the speed, but not in a systematic way hence conclusive observations were not possible. This could be due to the fact that the consolidant applied over the substrates had a certain thickness (about 1–2 mm), hence it was not so affected by the substrate at least in the first phases of sublimation.
- Camphene was shown to be too quick in sublimation for any use except for very short operations and/or very cold climates. When mixed with CDNOL (mix M3), camphene provided an only apparent acceleration of sublimation, as the camphene fraction was lost early and afterwards the rest of the material sublimed according to its own speed, without any particular benefit in terms of time necessary for the total disappearance of the consolidant from the surface.
- Menthol’s behaviour was influenced by the very low melting point of the product used, which makes it suitable especially for cold climates. It proved to be quicker in subliming than CDD, although in the same order of magnitude. When mixed with other VBM such as in M5 (with CDNOL) and M7 (with CDD) it was able to accelerate the sublimation. In particular, M7 was found to be the fastest among all the consolidants investigated, disappearing in some days over non-porous substrates at 20°C.
- CDNONE resulted a very interesting consolidant, as despite its similarity with CDD in terms of melting temperature, it is slower in sublimating than CDD but much faster than CDNOL.
- The mixes of CDD and CDNOL (M1 and M8) exhibited an intermediate sublimation behaviour, although also in these cases the sublimation occurred first for CDD and then for CDNONE.
- The mixes of CDNONE and CDNOL (M11 and M12) resulted very interesting, as they allowed to obtain intermediate sublimation speeds, proportional to the mass ratios of the components, and uniform mass loss with time. Over porous substrate, their sublimation was slower. The mix of CDD and CDNONE (M13) exhibited intermediate behaviour among the two pure VBM, too.
- The monitoring of the sublimation of the temporary consolidants for a long testing time is very important to highlight possible differences in speed owing to the separated loss of the single VBM.

The results obtained in this study showed that the investigated temporary consolidants may offer a wide range of tunable properties, allowing their use in a variety of climates and for different applications where short-term or long-term consolidation is necessary. However, more parameters will have to be investigated to pave the way to the application of these materials onsite, such as the viscosity, solidification speed, penetration depth in weathered substrates and cracks, shrinkage possibly damaging fragile substrates, presence of residues over the substrates after complete sublimation, behavior in a range of substrates representative of real buildings and artifacts. The viscosity of the melted VBM is particularly important, as it influences the capability of the products to penetrate cracks and re-aggregate the deteriorated artifacts, however such viscosity is expected to vary with time when the VBM is casted onto the artifacts, due to the difference of temperature between VBM and substrate, the thermal conductivity and cooling speed of the VBM, the temperature of the air, etc. These aspects will be the systematically evaluated in the future.

Moreover, it is noteworthy that a systematic assessment of the performance of temporary consolidants is a challenging task, as their purpose is somehow diffent from the purpose of ordinary consolidants such as TEOS, nanolime, hydroxyapatite, acrylic resins, etc. Ordinary consolidants are required to penetrate deep enough in the substrate and re-aggregate it in a compatible and durable way, hence a series of requirements in terms of effectiveness, compatibility and durability exist, which are accepted long since in the scientific community (e.g., penetration depth, absence of pore blocking effect, increase of the substrate’s cohesion/strength, limited aesthetic impact, etc.)^28–30^. On the contrary, no formal requirements exist for temporary consolidants. In fact, the concept of effectiveness for temporary consolidants depends on the targeted application, which can be to simply incorporate an object so to allow its handling and transportation, to shelter the substrate from water-based treatments or to support a thin layer. Depending on the application, for example, a deep penetration may be positive (for re-aggregation) or negative (for the sublimation time). Compatibility has a different meaning as well, as it basically consists in the absence of undesired chemical reactions, shrinkage or expansion of the VBM, residues remaining after complete sublimation. Finally, the meaning of durability is antithetical with respect to ordinary consolidants, as temporary consolidants must exhibit a lack of durability, i.e., they must be completely loss is a desired time. These observations arise the need of a wider discussion within the conservation and scientific community about the requirement of VBM and the relevant testing methods.

## Data Availability

The datasets used and/or analysed during the current study available from the corresponding author on reasonable request.
